# Emergency Department Frequent Users for Acute Alcohol Intoxication

**DOI:** 10.5811/westjem.2017.10.35052

**Published:** 2018-02-26

**Authors:** Lauren R. Klein, Marc L. Martel, Brian E. Driver, Mackenzie Reing, Jon B. Cole

**Affiliations:** Hennepin County Medical Center, Department of Emergency Medicine, Minneapolis, Minnesota

## Abstract

**Introduction:**

A subset of frequent users of emergency services are those who use the emergency department (ED) for acute alcohol intoxication. This population and their ED encounters have not been previously described.

**Methods:**

This was a retrospective, observational, cohort study of patients presenting to the ED for acute alcohol intoxication between 2012 and 2016. We collected all data from the electronic medical record. Frequent users for alcohol intoxication were defined as those with greater than 20 visits for acute intoxication without additional medical chief complaints in the previous 12 months. We used descriptive statistics to evaluate characteristics of frequent users for alcohol intoxication, as well as their ED encounters.

**Results:**

We identified 32,121 patient encounters. Of those, 325 patients were defined as frequent users for alcohol intoxication, comprising 11,370 of the encounters during the study period. The median maximum number of encounters per person for alcohol intoxication in a one-year period was 47 encounters (range 20 to 169). Frequent users were older (47 years vs. 39 years), and more commonly male (86% vs. 71%). Frequent users for alcohol intoxication had higher rates of medical and psychiatric comorbidities including liver disease, chronic kidney disease, ischemic vascular disease, dementia, chronic obstructive pulmonary disease, history of traumatic brain injury, schizophrenia, and bipolar disorder.

**Conclusion:**

In this study, we identified a group of ED frequent users who use the ED for acute alcohol intoxication. This population had higher rates of medical and psychiatric comorbidities compared to non-frequent users.

## INTRODUCTION

Frequent users of emergency departments (EDs) have been the subject of substantial research given the implications for resource utilization, healthcare costs, and ED crowding. 1–5 A unique subset of frequent ED users are those who present to the ED repeatedly for acute alcohol intoxication. 6–8 As ED visits for acute alcohol intoxication are increasing, 9 the burden of alcohol-related frequent users will be important to explore. Existing studies describing frequent ED users often cite alcohol-use disorders as a common comorbidity and a precipitant for their disproportionate utilization of emergency services. 1,2 Despite this established association, there is a paucity of data describing the encounters and individuals who frequently use the ED for alcohol intoxication, or the extent to which they use the ED for other reasons. The purpose of this study was to describe this population and their ED encounters.

## METHODS

This was a retrospective, observational, cohort study of ED patients presenting for acute alcohol intoxication from 2012 to 2016. It was approved by the institutional review board. The study hospital is a county ED with an annual volume of 100,000 visits and 7,000 visits for alcohol intoxication. The ED has a 16-bed area within the department that clusters all intoxication encounters. The purpose of this area is to treat patients who are in the department *for* intoxication at patients who ared to treat complicated medical or trauma patients who also happen to be intoxicated from alcohol. Patients are selected for treatment in this area at the discretion of triage nurses, paramedics (if arriving by ambulance), and the emergency physicians. All alcohol-intoxication encounters are seen in this particular area of the ED, but there is occasional overflow to other parts of the ED if these rooms are full. All patients who are treated in one of these rooms are entered into the electronic medical record (EMR) using the chief complaint “altered mental status.”

We included adults (>17 years old) if they presented to the ED for alcohol intoxication during the study period. These patients were identified using the EMR by querying for all visits where the chief complaint was “altered mental status ng for ir initial ED room was within the intoxication section of the ED. Patients were excluded if their breath alcohol concentration was zero. The variables for analyses were chosen a priori. We selected them if they were hypothesized to be relevant to the study population and if they were readily available in the EMR. A data analyst (trained in EMR data acquisition) who was blinded to the purpose of the study obtained the following variables without any manual chart abstraction: age, gender, race/ethnicity, insurance status, primary care physician, medical/psychiatric comorbidities, breath alcohol concentration, testing obtained (imaging, laboratory), chemical sedation administered, ED disposition, and length of stay. Additional data for each frequent user was manually abstracted from the chart by another investigator (MR); these included counts of ED visits that were not for alcohol intoxication, hospital admissions, and visits to a separate psychiatric services ED.

Multiple definitions for ED frequent users exist in the literature, ranging from 3–20 visits per 12-month period. 1,10 For this study, we elected to use the upper limit of this range and categorize an alcohol-related frequent user as greater than 20 visits for acute alcohol intoxication in the previous 12 months, in order to describe the highest-user cohort possible. Non-frequent users were those who did not meet this criterion.

After we identified the frequent-user cohort, we analyzed encounter characteristics for those with a frequent-user designation during that visit compared to those without. For analysis of patient characteristics and demographics, duplicate observations were excluded. The patient encounter that was retained for demographic analysis was the most recent encounter during the study period. For all comparisons, we calculated differences in means or proportions with associated 95% confidence intervals. We checked a subset of 20 charts to confirm accuracy of data abstraction.

**Population Health Research Capsule**

What do we already know about this issue?

Frequent users pose a unique challenge in emergency departments (ED), given their impact on resource utilization, healthcare costs, and their overall health considerations.

What was the research question?

The purpose of this study was to describe ED frequent users for alcohol intoxication and their ED encounters.

What was the major finding of the study?

Alcohol-intoxication frequent users had many medical/psychiatric comorbidities, and poor utilization of primary care.

How does this improve population health?

We intend the findings of this research to inform ED providers and community resource personnel to help them optimize care for this high-risk population.

## RESULTS

We identified 32,121 encounters meeting inclusion criteria (Figure), and there were 325 unique patients defined as frequent users for alcohol intoxication. These 325 patients represented 11,370 of the encounters during the study period. The median maximum number of encounters in a one-year period was 47 encounters (range 20 to 169) for acute alcohol intoxication.

During the five-year study period, frequent users used the ED for non-alcohol intoxication purposes a total of 3,812 times (median per patient = 11, range = 0–91), were admitted to the hospital a total of 4,960 times (median per patient = 9, range = 0–89), and used psychiatric, acute care services a total of 753 times (median per patient = 2, range 0–78). Additional patient characteristics and encounter characteristics are depicted in Table 1 and Table 2. Accuracy of data abstraction was 98%.

## DISCUSSION

Frequent users for alcohol intoxication are a unique subset of frequent ED users who merit attention given increasing numbers of alcohol-related visits nationally. 9 In this study, we identified 325 patients with 11,370 encounters for alcohol intoxication over a five-year period, where some individuals used the ED for alcohol intoxication more than 100 times in a year.

In this study, we identified several variables that differed for frequent users compared to non-frequent users. First, there were comparatively higher rates of medical and psychiatric comorbidities among alcohol-related frequent users. This finding reiterates the complexity of this population, and the fact that any of these “routine” visits have the potential for clinical decompensation and may require resources beyond the scope of simple observation for intoxication. We also identified differences in demographics (frequent users tended to be older, non-Caucasian, and male), as well as differences regarding health insurance status (frequent users were more often insured with government assistance such as Medicaid). In contrast, several variables were not different among the two groups; namely, diagnostic workups were similar between the groups, but interpretation of this finding is limited by practice patterns at our institution, where workups tend to be minimal for most alcohol-intoxication encounters.

Another important finding in this study was the low admission rate among frequent users (3%). While it is not unexpected that presentations for alcohol intoxication would result in low admission rates (as this is generally an uncomplicated chief complaint compared to other chief complaints), it does illustrate a potential barrier in caring for this population. In other studies describing frequent users for other general medical complaints, admission rates are reported to be as high as 40%.^3^ In those cases, interventions can be implemented as inpatients, and resources can be initiated during admissions. In the population we describe, since admissions are so uncommon, the responsibility may be on ED personnel to identify these patients, as they will not be addressed by an inpatient team.

In our cohort of alcohol-related frequent users, we identified some concerning features regarding primary care access and utilization. Less than half (49%) of the frequent-user population had primary care physicians, and only 4% were participants in a coordinated primary care program intended for the hospital’s greatest utilizers. We believe that this is an important gap in coverage for a very high-needs population. This finding also contrasts the general ED frequent-user literature, where most describe primary care access as over 90%. 1 Our institution does not appear to be identifying alcohol-related frequent users for primary care services as effectively as those who use the ED for other problems. Possible explanations for this gap in coverage could include a lack of readiness for healthcare accountability, or a struggle maintaining primary care relationships in the setting of ongoing substance abuse.

We were unable to determine the prevalence of important social stressors such as homelessness, employment, or government assistance (other than health insurance) in this cohort, but addressing these stressors in future will play an important role in assisting this population. Multiple social services interventions have been proposed for frequent ED users, such as case management and referral programs, but these have been shown to have variable rates of success. 7,11 One study conducted in our community investigated use of case management and demographic-specific housing referrals among 92 chronic inebriates. While the study found that the healthcare costs decreased pre vs. post intervention, ED visits did not decrease. 7

## LIMITATIONS

This study is subject to several limitations, including those inherent to a retrospective study design. We attempted to minimize this bias by using standardized methods for data collection research. Second, we present data from a single center, which may not be generalizable to other EDs, especially for EDs that do not see large volumes of alcohol-intoxication visits. We do believe, however, that many of our findings coincide with existing literature describing other populations of ED frequent users, thus supporting our results. It is also likely that there are other important variables describing this population (such as other comorbidities) that were not explored in this study.

Another potential limitation was our definition of frequent users. We elected to use a cutoff of 20 visits per 12 months to define our frequent users based on previous literature. The intent of using this particular cutoff was to capture the highest-user group possible, but it is possible that had we used other cutoff definitions, our results would be different. Finally, there is the potential for selection bias in this sample, as we focused our search query on alcohol-intoxication visits in a specific area of our ED, rather than the entire ED. This was, however, the most practical means to fulfill the goals of this study, which was to ensure that the encounters we describe were those *for* alcohol intoxication, rather than encounters for a primary medical or traumatic purpose where the patient was also intoxicated from alcohol.

## CONCLUSION

There are patients who frequently use the ED for acute alcohol intoxication, and this group of “alcohol-related” frequent users has not been previously reported. We identified that this frequent-user population has higher rates of medical comorbidities and psychiatric comorbidities compared to non-frequent users. This population was also found to have relatively poor access to primary care (less than 50%). We intend the findings of this report to be hypothesis-generating for future work regarding how to target this population.

## Figures and Tables

**Figure:**
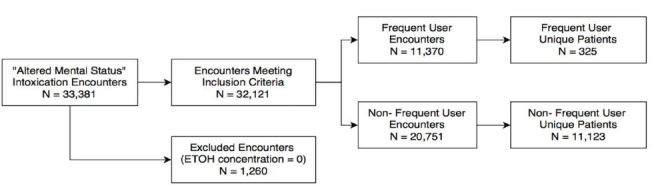
Patient inclusion and exclusion in study examining frequency of emergency department use by those with acute alcohol intoxication.

**Table 1:** Patient characteristics for alcohol-related frequent vs. non-frequent users.

Patient variable	Frequent user (n=325)	Non-frequent user (n=11,123)	Difference (95% CI)
Age (mean years)	47	39	8 (95% CI [6–9])
Gender (% male)	281 (86%)	7880 (71%)	16% (95% CI [11–19])
Race/Ethnicity			
Caucasian	101 (31%)	6,407 (58%)	−27% (95% CI [−32 to −22%])
African/African-American	102 (31%)	2,374 (21%)	10% (95% CI [5–15%])
Native American	108 (33%)	945 (9%)	24% (95% CI [19–29%])
Hispanic	10 (3%)	718 (6%)	−3% (95% CI [−5 to −1%)
Asian	2 (1%)	161 (1%)	0 (95% CI [−1 to 1%])
Primary care physician	161 (49%)	3105 (28%)	22% (95% CI [16–26])
Coordinated primary care services	14 (4%)	67 (0.6%)	3% (95% CI [1–6])
Insurance			
No insurance	68 (21%)	4110 (37%)	−16% (95% CI [−21 to −11])
Medicaid	87 (27%)	1248 (11%)	16% (95% CI [11–21%])
Medicare	37 (11%)	920 (8%)	3% (95% CI [0–6%])
Medical assistance	74 (23%)	1247 (11%)	12% (95% CI [7–17%])
Private insurance	26 (8%)	2148 (19%)	−11% (95% CI [−14 to −8%])
Medical comorbidities			
Liver disease	89 (27%)	611 (6%)	21% (95% CI [16–26])
Chronic kidney disease	35 (11%)	440 (4%)	7% (95% CI [4–10])
Ischemic vascular disease	22 (7%)	179 (2%)	5% (95% CI [2–8])
COPD	30 (9%)	184 (2%)	7% (95% CI [4–10])
History of TBI	62 (19%)	342 (3%)	16% (95% CI [12–20])
Dementia	30 (9%)	170 (2%)	7% (95% CI [4–10])
Psychiatric comorbidities			
Schizophrenia	40 (12%)	320 (3%)	9% (95% CI [5–13])
Bipolar disorder	67 (21%)	778 (7%)	14% (95% CI [10–18])

*CI*, confidence interval; *COPD*, chronic obstructive pulmonary disease, *TBI*, traumatic brain injury.

Patient characteristics calculated using a single encounter per patient (excluding duplicate encounters).

**Table 2:** Encounter characteristics for alcohol-related frequent users vs. non-frequent users.

Encounter variable	Frequent user encounter (n=11,370)	Non-frequent user encounter (n=20,751)	Difference (95% CI)
Initial BAC (mean mg/dl)*	256	221	35 (95% CI 33–37)
Admitted to hospital	340 (3%)	627 (3%)	0% (95% CI 0–1)
ICU admissions	109 (1%)	189 (1%)	0% (95% CI 0–1)
Laboratory testing	725 (6%)	1523 (7%)	−1% (95% CI −1 to 0)
CT performed	434 (4%)	1309 (6%)	−2% (95% CI −3 to −2)
Chemical sedation	3957 (35%)	8987 (43%)	−8% (95% CI −10 to −7)
Length of stay (mean minutes)	470	482	−12 (95% CI −17 to −7)

*BAC*, blood alcohol concentration; *CI*, confidence interval; *ICU*, intensive care unit; *CT*, computed tomography.

Encounter characteristic calculated using all encounters, including multiple encounters per patient.

* BAC was performed on 100% of patients.

## References

[1] LaCalle E, Rabin E (2010). Frequent users of emergency departments: the myths, the data, and the policy implications. Ann Emerg Med.

[2] Doupe MB, Palatnick W, Day S (2012). Frequent users of emergency departments: developing standard definitions and defining prominent risk factors. Ann Emerg Med.

[3] Locker TE, Baston S, Mason SM, Nicholl J (2007). Defining frequent use of an urban emergency department. Emerg Med J.

[4] Pines JM, Asplin BR, Kaji AH (2011). Frequent users of emergency department services: gaps in knowledge and a proposed research agenda. Acad Emerg Med.

[5] Leporatti L, Ameri M, Trinchero C (2016). Targeting frequent users of emergency departments: Prominent risk factors and policy implications. Health Policy.

[6] Whiteman PJ, Hoffman RS, Goldfrank LR (2000). Alcoholism in the emergency department: an epidemiologic study. Acad Emerg Med.

[7] Thornquist L, Biros M, Olander R (2002). Health care utilization of chronic inebriates. Acad Emerg Med.

[8] Biros MH (1996). The frequency of unsuspected minor illness or injury in intoxicated patients. Acad Emerg Med.

[9] Mullins PM, Mazer-Amirshahi M, Pines JM (2017). Alcohol-Related Visits to US Emergency Departments, 2001–2011. Alcohol.

[10] Curran GM, Sullivan G, Williams K (2003). Emergency department use of persons with comorbid psychiatric and substance abuse disorders. Ann Emerg Med.

[11] Althaus F, Paroz S, Hugli O (2011). Effectiveness of interventions targeting frequent users of emergency departments: a systematic review. Ann Emerg Med.

